# Ultradian Rhythms in the Transcriptome of *Neurospora crassa*

**DOI:** 10.1016/j.isci.2018.11.012

**Published:** 2018-11-10

**Authors:** Bharath Ananthasubramaniam, Axel Diernfellner, Michael Brunner, Hanspeter Herzel

**Affiliations:** 1 Institute for Theoretical Biology, Humboldt-Universität zu Berlin, 10115 Berlin, Germany; 2 Charité Universitätsmedizin, 10117 Berlin, Germany; 3 Biochemistry Center, Universität Heidelberg, 69120 Heidelberg, Germany

**Keywords:** Biological Sciences, Chronobiology, Transcriptomics

## Abstract

In many organisms, the circadian clock drives rhythms in the transcription of clock-controlled genes that can be either circadian (∼24-hr period) or ultradian (<24-hr period). Ultradian rhythms with periods that are a fraction of 24 hr are termed *harmonics*. Several harmonic transcripts were discovered in the mouse liver, but their functional significance remains unclear. Using a model-based analysis, we report for the first time ∼7-hr *third harmonic* transcripts in *Neurospora crassa*, a well-established fungal circadian model organism. Several third harmonic genes are regulated by female fertility 7 (FF-7), whose transcript itself is third harmonic. The knockout of circadian output regulator CSP1 superimposes circadian rhythms on the third harmonic genes, whereas the knockout of stress response regulator MSN1 converts third harmonic rhythms to second harmonic rhythms. The 460 ∼7-hr genes are co-regulated in two anti-phasic groups in multiple genotypes and include kinases, chromatin remodelers, and homologs of harmonic genes in the mouse liver.

## Highlights

• Coexisting harmonic ∼7-hr and circadian rhythms in fungal clock model organism
• Knockout of output regulator CSP1 imposes circadian rhythms over ∼7-hr rhythms
• Third harmonic rhythms are a part of key cellular processes and mediated by FF-7
• 7-hr genes are co-regulated in two anti-phasic clusters across genotypes and laboratories

## Graphical Abstract

**Figure:**
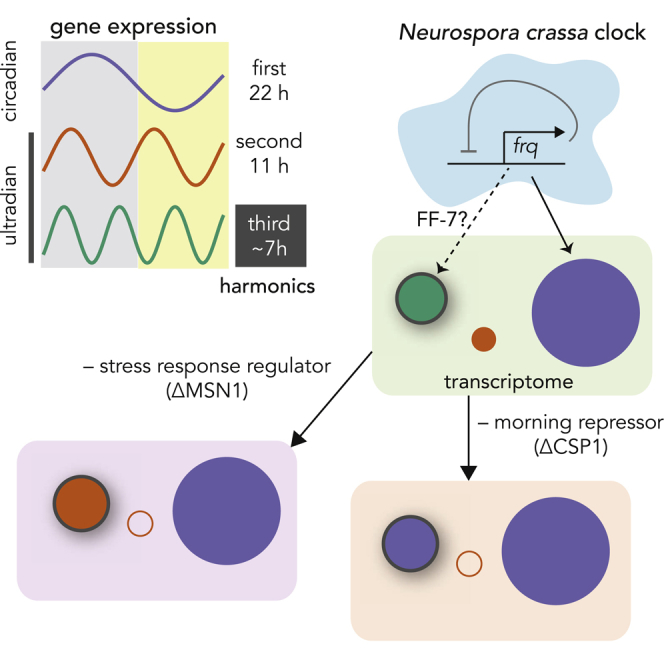

## Introduction

Many organisms evolved a circadian timekeeper to adapt to the 24-hr day-night cycle on earth. This timekeeper or circadian clock can be found among plants, animals, and fungi and is thought to have evolved independently multiple times (Liu and Bell-Pedersen, 2006, Dunlap and Loros, 2017). The conserved basis for circadian rhythm generation is delayed negative feedback loops involving either purely translational or a combination of transcriptional and translational processes (Bell-Pedersen et al., 2005). For example, within the core clock of the fungus *Neurospora crassa*, the positive regulator white collar complex (WCC), a heterodimer of GATA-type transcription factors (TFs) *wc-1* and *wc-2*, drives transcription of the negative element frequency (*frq*) that after transcription, translation, and successive phosphorylations suppresses the activity of WCC (Larrondo et al., 2015, Hong et al., 2008). Eventually, further phosphorylation eliminates the interaction of WCC and FRQ allowing the cycle to begin again. The core circadian clock then regulates many physiological processes within the organism to achieve the necessary temporal adaptation and anticipation.

The primary output of the core clock is rhythmic transcription of a group of genes called *clock-controlled genes* (ccgs) via one or more TFs that are part of the core clock. These ccgs are classified as *circadian*, if they have a period close to 24 hr. However, these transcript rhythms can have a period shorter than 24 hr, in which case they are classified as *ultradian*. In particular, when the ultradian rhythms have a period that is a fraction of 24 hr (circadian period), the rhythms can be considered harmonics. For example, 12-hr rhythms are second harmonic (half the circadian period) and 8-hr rhythms are third harmonic (a third of the circadian period). Such scenarios are of interest for two reasons. First, a harmonic rhythm is also periodic over a 24-hr interval. Second, the relationship between the period lengths is suggestive of regulation by the circadian clock (that has a 24-hr period).

Hughes et al. (2009) first discovered such harmonic transcriptional rhythms in the mouse liver and showed that the ca. 300 harmonic genes were produced only in the *in vivo* context through the interaction of multiple rhythmic inputs. More recently, such harmonic rhythms have also been observed at the proteomic and metabolomic levels (Krishnaiah et al., 2017). Outside of terrestrial model organisms, approximately 12-hr (second harmonic) rhythms have been shown to exist as part of circa-tidal clocks that function independently of the circadian clock (Zhang et al., 2013, Zantke et al., 2013). Zhu et al. (2017) recently identified 12-hr harmonic transcriptional rhythms in the liver of mice lacking a clock (i.e., in *Bmal1* knockout mice) and conjecture from gene conservation that these second harmonics might be generated by remnants of an ancestral circa-tidal clock. Nevertheless, there have been no studies that have identified harmonic ccgs in circadian model organisms other than in mammals.

In this work, we report for the first time third harmonic ∼7-hr rhythms in the transcriptome of *N*. *crassa*, the model clock organism in the fungal kingdom. These nearly 500 third harmonic genes include TFs, kinases, and chromatin remodelers and four classes of genes that are conserved between fungi (*Neurospora*) and mammals (mice), i.e., expressed as harmonics in both organisms. Although the harmonics do not appear to be directly driven by known circadian TFs, they are affected by the morning repressor, CSP1, in whose absence the third harmonic genes acquire a superimposed circadian rhythm. Furthermore, loss of the stress response transcription factor MSN1 turns the ∼7-hr third harmonic rhythms into 11-hr harmonic rhythms. The TF female fertility 7 (FF-7), whose transcript itself is third harmonic, likely drives downstream third harmonic rhythms in a significant fraction of genes.

## Results

### Third Harmonic Rhythms with Near-7-hr Period Are Widespread in *N*. *crassa*

**Figure 1:**
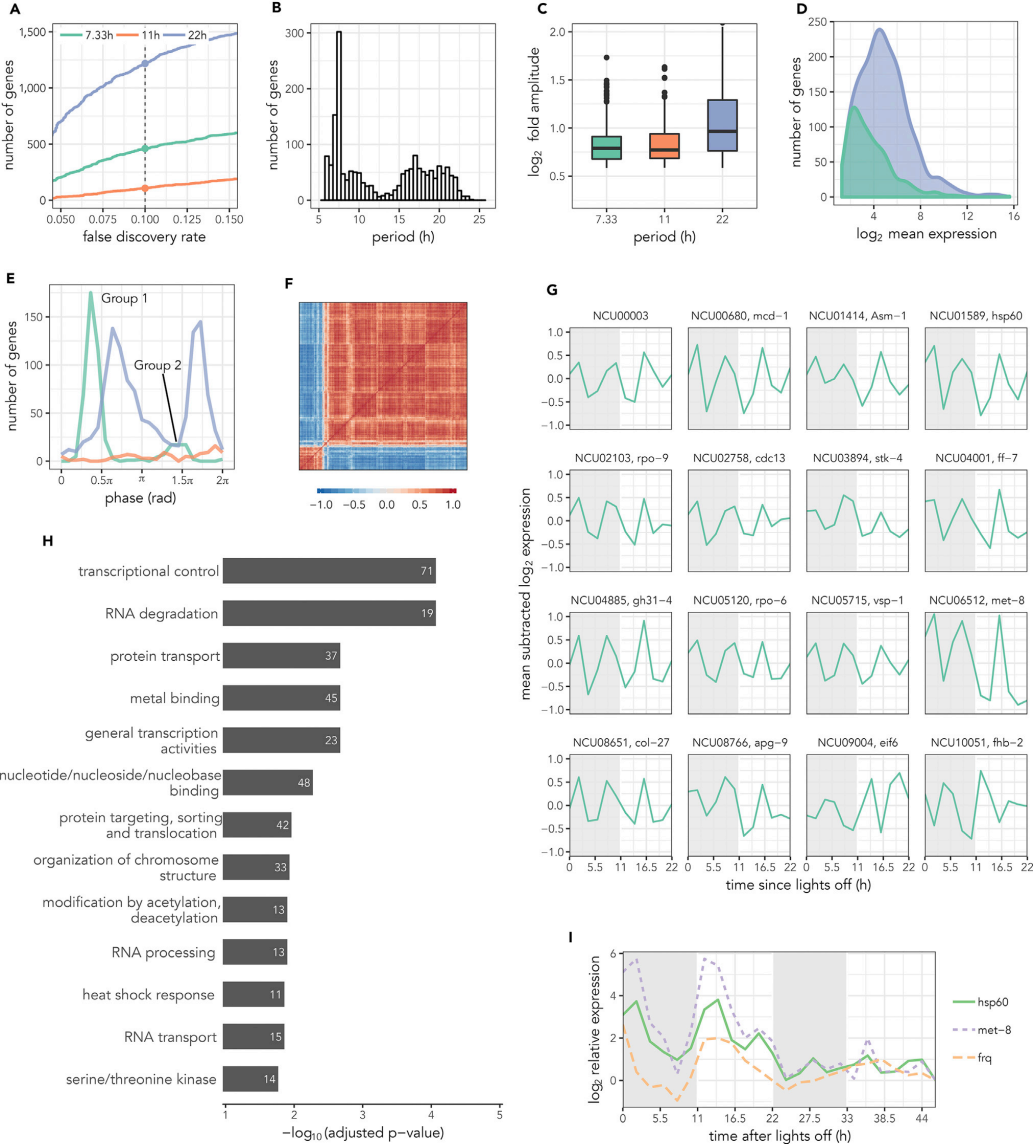
Quantification of 7-hr Transcripts in *N*. *crassa* (A–I) (A) The number of rhythmic genes at each period identified by the model-selection-based procedure at different false discovery rate thresholds. The color coding for the different periods is maintained throughout the figure. (B) The distribution of periods of the rhythmic genes as estimated by the ARSER (Wu et al., 2016). The distribution of peak-to-peak fold amplitudes (C), mean expression level (D), and phases (E) of the different rhythmic genes quantified by the model-selection-based procedure. The second harmonic genes have been omitted in (D) for clarity. (F) Correlation-metric-based hierarchical clustering of the mean-subtracted expression profiles of the third harmonic genes. (G) Transcript profiles of selected third harmonic genes, such as transcription factors, kinases, and chromatin remodelers. (H) Top functional categories (FDR<0.05) enriched in the third harmonic genes using FungiFun2 (Priebe et al., 2015). (I) Verification of 7-hr rhythms in two candidate genes using quantitative real-time PCR over a 2-day time course with 2-hr time resolution. The results for *met-8* and *hsp60* in a WT strain are shown. The circadian clock gene *frq* is included as a positive control of the quantitative real-time PCR analysis. Expression levels relative to the last time point are plotted for each gene. See also Figures S1 and S2 and Transparent Methods.

The model-selection-based procedure (see Transparent Methods and Figure S1A) found 1,784 rhythmic genes (with 7.33-, 11-, or 22-hr period) at a false discovery rate (FDR) of 0.1 among 7,212 expressed genes in the wild-type (WT) strain in the dark after 11 hr:11 hr light-dark (LD) entrainment. Consistent with earlier analyses of these data (Sancar et al., 2015b), we found 1,218 circadian (22 hr) transcripts (Figure 1A). Note that *N*. *crassa* has an intrinsic period of ∼22.5hr and hence we used 22 hr as the circadian period. Surprisingly, we discovered 460 and 106 genes (Table S1) at the third and second harmonics, respectively. The relative proportion of these three oscillation periods was robust to the choice of FDR threshold. We also verified that these oscillation periods indeed exist using an independent method called ARSER from the MetaCycle package (Wu et al., 2016) (Figure 1B). Owing to the preponderance of third harmonic rhythms when compared with the second harmonic rhythms, we focus on such harmonic genes in the rest of this work.

The third harmonic rhythms have a peak-to-peak amplitude (Figure 1C) and magnitude (mean) of expression (Figure 1D) comparable to the circadian rhythms. The third harmonic genes were clustered in two anti-phasic groups (Figure 1E), much like the circadian ccgs as previously reported (Sancar et al., 2015b). In addition, correlation-based hierarchical clustering of the expression profiles of the third harmonic genes split the gene set into two anti-phasic clusters with each cluster having highly similar profiles (Figure 1F). The expression phase-based and correlation-based clusters matched precisely, and we henceforth refer to the clusters as Group 1 (with 392 genes) and Group 2 (with 68 genes) (Figure 1E).

We then attempted to confirm the existence of the harmonic rhythms in two ways. First, we repeated the original WT high-throughput sequencing on fresh cultures under the same conditions and analyzed the data (Figures S2A and S2B). We were able to observe third harmonic gene expression in the gene set in two anti-phasic clusters (see Figures S2C–S2F), although all rhythms (circadian and harmonic) had smaller amplitudes and hence fewer were statistically detected. To verify that the rhythms exist but were only not detected in that sequencing run, we analyzed two selected genes using a different approach, i.e., quantitative real-time PCR. We chose the two genes (*met-8* and *hsp60*) based on four criteria: (1) the peak-to-trough amplitude of the 7-hr transcripts is at least 2-fold; (2) the magnitude of expression is greater than 32 a.u., a cutoff that selects robustly expressed genes; (3) the 7-hr rhythm is also observed in the ΔCSP1 strain (discussed later); and (4) the gene is a known annotated gene. We also included the clock gene *frq* for quality control of the samples. The 7-hr rhythms were clearly reproducible in the fresh WT cultures (Figure S2G) with the rhythms showing at least a 2-fold amplitude assumed in the selection criteria. Second, we empirically estimated the probability of observing 460 third harmonic genes by random chance (by randomly shuffling the time labels) to be less than 8% (Figure S2H), which is below our design FDR threshold.

We next tested if the ∼7-hr rhythms persist beyond 1 day and hence are self-sustained. We performed the WT experiment over a longer time span of 46 hr and measured the expression of the two selected genes, *met-8* and *hsp60*, and *frq* as control using quantitative real-time PCR. The rhythms with third peaks of activity a day did persist beyond 1 day, albeit with strong dampening of the rhythms after day 1 (Figure 1I). The rhythms in *met-8* and *hsp60* were nevertheless in phase, as we expected from the other datasets. To our surprise, long-term monitoring (over multiple days) of the promoters of these two genes using a luciferase assay (Cesbron et al., 2013) in solid media (as opposed to liquid media for all the previous experiments) yielded oscillations in several clones that were not ultradian (Figure S2I).

The third harmonic genes included several kinases, chromatin remodelers, and those involved in regulation of transcription, heat shock response, protein transport, and RNA processing, according to functional categorization (Figures 1G and 1H, Table S2). These also included 17 of 132 expressed putative and known TFs (Colot et al., 2006), such as *ff-7*, *ada-3*, *tah-3*, *col-27,* and *asl-1*. Examples of kinases in this gene set are *stk-4*, *ste-7*, *prk-4,* and *prk-8*, and the histone deacetylase *hda-4*. Surprisingly, we found *frh*, the interaction partner of the *Neurospora* core clock component *frq*, also among the third harmonic genes.

Several hundred genes are conserved among eukaryota, and the mammalian and fungal circadian clock mechanisms are highly similar (Liu and Bell-Pedersen, 2006). Therefore, we looked for conservation between the third harmonic genes and the harmonics (both 8- and 12-hr period) in the mammalian liver transcriptome (Hughes et al., 2009) using HomoloGene (Homologene [Internet], 2017). Unexpectedly, we found four families of genes that were conserved (Table 1): subunits of the RNA polymerase II, translation initiation factors, histone deacetylases, and heat shock proteins, one of which we verified using quantitative real-time PCR.

**Table 1:** Conserved Harmonic Genes in Mammals and Fungi

*N*. *crassa*	Gene Family	*Mus musculus*
*rpo-6*, *rpo-9*	DNA-directed RNA polymerase II	*Pol2ri*
*eif3a*, *eif3e*, *eif2β*, *eif5b*, *eif6*	Eukaryotic translation initiation factors	*Eif1a*, *Eif3s10*, *Eif2ak3*
*hsp60*, *hsp70-5*, *hsp70-6*, *hsp88*	Heat shock proteins	*Hspa1b*, *Hspa5*
*hda-4*	Histone deactylases	*Hdac1*

### Generation of Harmonics

**Figure 2:**
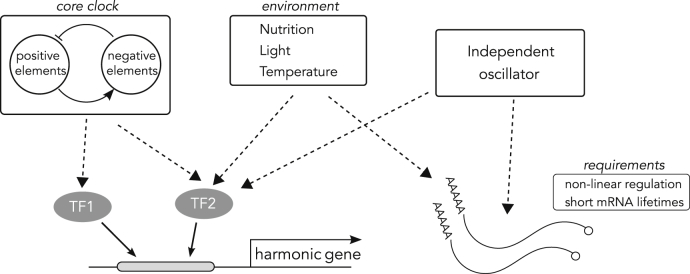
Possible Mechanisms for Generation of Harmonics The harmonics can be generated by combinatorial regulation of periodic factors that are outputs of the circadian clock or periodic environmental inputs, such as light-dark cycles, or by an independent oscillator. The transcripts also need to have sufficiently low stability in order for the harmonics to be observable. See also Figure S3.

The observed harmonics can theoretically originate from three possible mechanisms summarized in Figure 2. First, multiple circadian regulators (e.g., TFs, RNA-binding proteins) with appropriate phases driving transcription of an output gene transcriptionally and/or post-transcriptionally can generate harmonics. For example, Westermark and Herzel (2013) showed that two circadian TFs with anti-phasic transcriptional activity and binding cooperativity can generate second harmonic 12-hr rhythms. Similarly, three circadian TFs with appropriate phases and strengths might produce third harmonic rhythms, but theoretically the regulators need not only be transcriptional in nature.

Second, the harmonics can be purely generated by one or more periodic environmental inputs, such as LD cycles and nutritional inputs, driving a resonating passive gene regulatory network. Third, these harmonics might be the output of an oscillator independent of the circadian system. The interaction between the independent oscillator and the circadian clock can then result in mode locking and a harmonic relationship of the periods.

### CSP1 Suppresses Circadian Rhythms in Third Harmonic Genes

We first tested the hypothesis whether these third harmonic rhythms might be driven by the 11 hr:11 hr LD cycles. To this end, we checked if these genes acutely respond to lights on in a functional assay (Sancar et al., 2015a). However, only a small fraction of genes (29) responded to light over a span of 2 hr (Figure S3A), which included only two putative TFs, *asl-1* and *vad-2*, suggesting that light is unlikely to play a direct role in generating these harmonics.

Next, we examined whether these third harmonic rhythms are an output of the circadian clock. Hence, we first examined if these genes were direct targets of known circadian output regulators using available chromatin immunoprecipitation sequencing (ChIP-seq) datasets (Table S4) for TFs: WCC, CSP1, RCO1, and SUB1 (Smith et al., 2010, Sancar et al., 2011, Sancar et al., 2015b, Sancar et al., 2015a) (Figure S3B). Incidentally, FF-7, an interaction partner of SUB1, had significantly enriched (*p* = 4.1×10^−3^, Fisher's exact test) binding sites in the vicinity of third harmonic genes, and moreover, the transcript of FF-7 is also third harmonic (Figure 1G).

**Figure 3:**
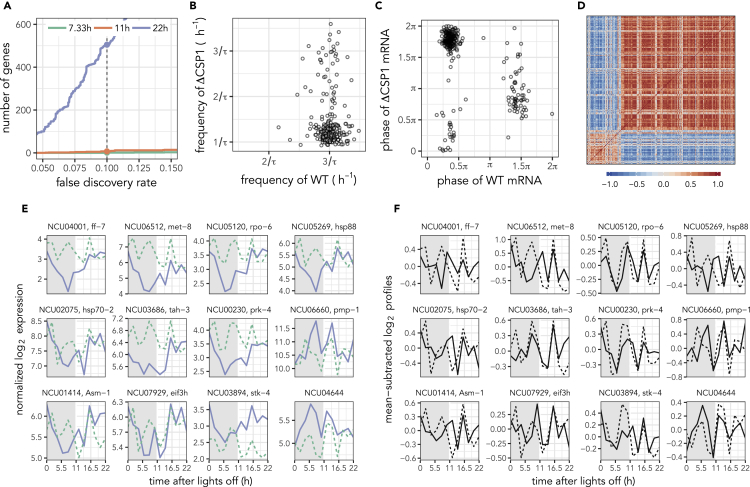
Superimposed Circadian Rhythms in the Third Harmonic Genes in the ΔCSP1 Strain (A) The number of rhythmic genes at each harmonic for different FDR thresholds. (B) The change in frequency (estimated using ARSER) of the third harmonic genes between the wild-type (WT) strain and the ΔCSP1 strain. (C) Comparison of the phases of the third harmonics genes between the WT and ΔCSP1 strains at their respective periods. (D) Pairwise correlation of the gene expression profiles of the third harmonic genes in the ΔCSP1 strain arranged according to the same clustering in Figure 1F. (E) The transcript profiles for selected genes are shown in the WT (dashed) and ΔCSP1 (solid) strains. Colors represent the oscillation periods as in (A). (F) The transcript profiles for the same genes in (E) after subtraction of the induced circadian rhythms in the ΔCSP1 strain (solid) with the profile in the WT strain (dashed) as reference. See also Figure S3.

Although we did not find an overrepresented circadian TF in the ChIP-seq data, we decided to disrupt a large fraction of clock-controlled genes to gauge if that would perturb the third harmonic rhythms. The morning repressor CSP1 is known to drive more than a 1,000 downstream ccgs (Sancar et al., 2015b), and unlike the main circadian output TF WCC (a core clock component), the knockout of CSP1 does not disrupt the core circadian clock. Hence, we quantified the effect of knocking out the morning repressor CSP1 on the transcriptome of *Neurospora*. Our model-selection-based approach detected only 495 circadian ccgs and less than 10 harmonic genes in the ΔCSP1 knockout strain (Figure 3A). Nonetheless, the expression of 1,105 circadian genes identified in the WT (Figure 1A and Table S1) were enriched for circadian (22 hr) components in ΔCSP1 strain (Figure S3D) and the circadian rhythms in these genes are evident under suitable phase ordering (Figure S3C) consistent with the original study (Sancar et al., 2015b).

Since we did not find any harmonic rhythms in the ΔCSP1 background, we wondered whether the third harmonic genes had lost their rhythmicity. The model selection approach we used assigns the “best” period (circadian versus harmonic) to each gene in the transcriptome. In other words, genes are assigned to only one of the three periods. In the ΔCSP1 strain, almost all the third harmonic genes in the WT strain were classified as circadian genes with the rest still classified as third harmonic (Figure 3B). The original set of third harmonic genes was also enriched for the circadian rhythmic component in the ΔCSP1 strain (Figure S3D). Furthermore, the circadian rhythms of third harmonic genes in ΔCSP1 still fall into two anti-phasic groups (π radians apart) like in the WT (Figure 3C) matching Groups 1 and 2. The expression patterns of genes within Group 1 and Group 2 (in Figure 1F) remain highly similar to those seen in the genewise cross-correlation of expression profiles (Figure 3D).

The change in the rhythms of the original third harmonic genes in Figure 1G is evident in Figure 3E. Although the circadian expression pattern that caused our model selection method to classify these genes as circadian is clear, a third harmonic rhythm component also appears to remain in several genes. When the circadian rhythm component was subtracted from the ΔCSP1 expression profiles in these genes, the residual third harmonic rhythm matched the third harmonic rhythms in the WT (Figures 3F and S3E) quite well. In other words, in the ΔCSP1 strain, the third harmonics genes acquire a circadian rhythm that is superimposed on the third harmonic rhythm seen in the WT.

### The Knockout of MSN1 Converts Third Harmonic Rhythms to Second Harmonic Rhythms

Next, to identify potential mechanisms involved in the generation of these third harmonic rhythms, we returned to the functional enrichment we performed earlier (Table S3). The most highly enriched specific category was serine/threonine kinases. In addition, we performed a KEGG pathway enrichment (Kanehisa et al., 2017) and found that RNA degradation and the yeast mitogen-activated protein kinase (MAPK) signaling pathways were highly enriched (Table S2). In particular, in our search for downstream TFs involved in either pathway, we found MSN1, a cutinase G-box-binding protein and ortholog of yeast MSN2p stress response TF. MSN1 was activated by a third harmonic protein kinase A (NCU06240), and its transcript had a third harmonic rhythm that was just outside statistical significance (adjusted p = 0.14). Conveniently, this TF was experimentally accessible, and its knockout has no effect on the core circadian clock as measured by conidiation (except for a reduced growth rate) (Zhou et al., 2018). We therefore decided to test in a genome-wide manner whether ΔMSN1 did indeed not have an effect on clock output, including the third harmonic rhythms.

**Figure 4:**
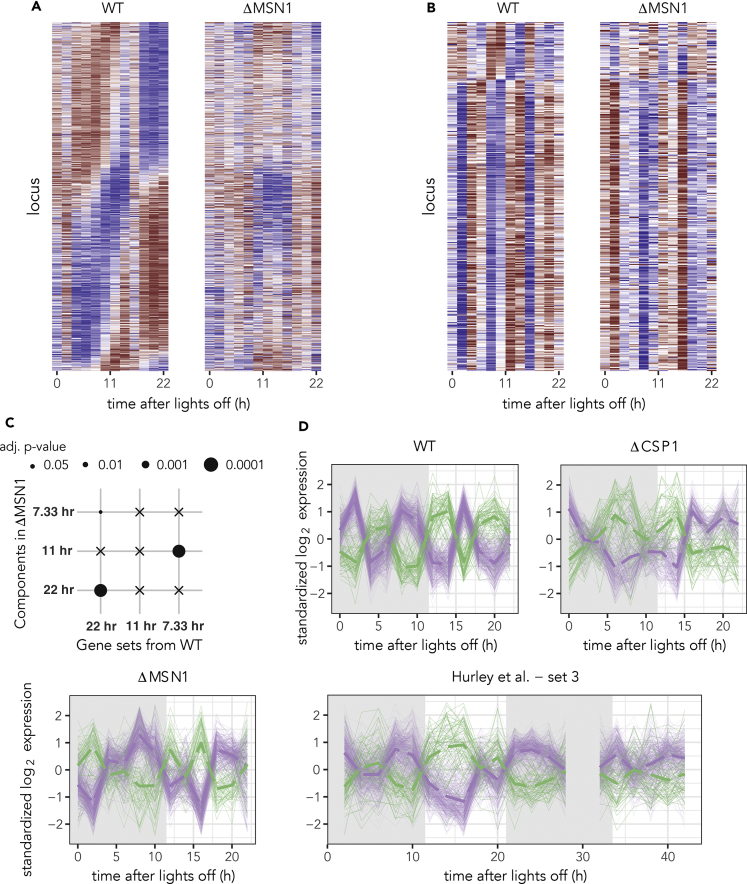
MSN1-Dependent Regulation of Third Harmonic Rhythms and Co-regulation of Anti-phasic Genes (A–D) Heatmaps comparing the expression of the circadian (A) and third harmonic genes (B) (from Figure 1) in the WT and ΔMSN1 strains. The genes are sorted according to their phases in the WT, and a common scale is used for all heatmaps. (C) Competitive gene set testing (Wu and Smyth, 2012) of the different harmonic components in the ΔMSN1 strain within the rhythmic sets identified in the WT strain. Adjusted p-values are rounded up to the four levels shown, and crosses represent no significance (>0.05). (D) The standardized (*Z* score) expression profiles of the Group 1 (violet) and Group 2 (green) genes in the different genotypes and in the WT dataset from Hurley et al. (2014). The average of the standardized profiles in each group is shown as thick dashed lines. Missing time points were omitted from the plots. See also Figures S4 and S5.

Model-selection-based classification of the rhythmic genes predominantly identified rhythms with 22- (circadian) and 11-hr (second harmonic) periods (Figures S4A, 4A, and 4B). The circadian (22 hr) transcriptome (from the WT) remained largely intact in the ΔMSN1 strain (Figure 4A). In particular, we observed statistically significant enrichment of circadian rhythms under ΔMSN1 in the circadian genes identified in the WT (Figure 4C), and vice versa (Figure S4C). Thus, there is significant overlap between the circadian genes. Nonetheless, the phases of the genes appeared to be shifted (Figure 4A), such that reordering the genes by phase in ΔMSN1 makes the circadian rhythms more pronounced (Figure S4B).

Interestingly, the classification found almost no third harmonic rhythms like in the ΔCSP1 strain (Figure S4A). Deeper investigation revealed that all the third harmonic genes had assumed a 11-hr (second harmonic) period (Figure 4B). This was statistically supported by the enrichment of second harmonic components in the ΔMSN1 strain among the WT third harmonic genes (Figure 4C) and complementarily, the enrichment of third harmonic components in the WT strain among the ΔMSN1 second harmonic genes (Figure S4C). Furthermore, the two groups of genes (Group 1 and Group 2) continued to be co-expressed in anti-phase. That is, loss of MSN1 either directly or indirectly disrupts one of the rhythmic factors involved in harmonic generation, as we described in Figure 2.

### Two Phase Groups of 7-hr Genes Are Discordantly Co-Regulated under a Variety of Conditions

As we explained in the previous sections, we observed complex rhythmic phenomena in the 460 third harmonic genes under different genotypes. The one consistent feature in all the data was the anti-phasic regulation of Group 1 and Group 2 genes or, to rephrase it more generally, discordant co-expression of these two groups of genes. This is clearly evident from the time courses of the two groups of genes (Figures 4D and S5A) and even more so in the pairwise correlation matrices of the expression profiles (Figure S5B).

To test this proposition, we analyzed another recently published high-throughput sequencing study of the *N*. *crassa* transcriptome in an independent laboratory (Hurley et al., 2014). This dataset slightly differs from the datasets we generated in that the *Neurospora* cultures were grown in constant light conditions (not under LD cycles) and then measured in the dark. The data were collected every 2 hr over a time span of 48 hr to produce three separate time courses (sets 1, 2, and 3). When we applied our model-selection-based classification, we did not find any third harmonic rhythm in any of the sets, but did find circadian rhythms as in the original study. Confirming this conclusion, circadian rhythms were enriched in the Hurley et al. (2014) dataset among the WT circadian genes (Table S1). However, we did find statistically significant enrichment of third harmonic rhythms in one of the three WT third harmonic gene sets (adjusted p-values were 0.63, 0.35, and 0.02 for sets 1, 2, and 3). Ultradian (∼7–8 hr) rhythms with a variable baseline are visible in this one dataset (Figure 4D).

We then proceeded to consider the expression patterns of the two anti-phasic groups of third harmonic genes (Group 1 and Group 2). We observed that first there was indeed some kind of noisy oscillation in these genes, especially in set 3 from Hurley et al. (2014). Second, Group 1 and Group 2 genes were co-expressed and discordant both from the expression profiles (Figures 4D and S5A) and from the pairwise gene expression correlation matrices (Figure S5B).

## Discussion

We discovered ultradian ∼7-hr rhythms in the *N*. *crassa* transcriptome for the first time using our model-selection-based approach for the identification of harmonics. Although the time course spanned only a single circadian period of 22 hr, it included three cycles of the 7-hr third harmonic rhythms sampled every 2 hr. This is sufficient to have confidence in statistically identifying these rhythms (Hughes et al., 2009). Nonetheless, we performed a new 46-hr (two circadian periods) time course experiment and established using a complementary method (quantitative real-time PCR) that the selected third harmonic genes do persist beyond 1 day, albeit with severely dampened rhythm amplitudes. It is in general difficult to determine from a population measurement if this dampening is a single cell (loss of rhythms) or population effect (desynchronization).

Our model-selection-based approach has better precision-recall performance than the standard method (Wichert et al., 2004), although there is some loss of power due to classification of rhythms strictly into one of the harmonics. We excluded the possibility that these rhythms are an analysis artifact, such as normalization, by confirming these profiles using both alignment-free (Kallisto [Bray et al., 2016]) and alignment-based approaches (STAR [Dobin et al., 2013] + featureCounts [Liao et al., 2014]). In addition, we confirmed the presence of third harmonics in three independent experiments (two repeated WT and one ΔCSP1 time series) in liquid cultures in at least one of two different methods (quantitative real-time PCR and RNA sequencing). However, we were unable to observe third harmonics in solid *Neurospora* cultures using luciferase reporter assays, although we observed oscillations in several clones. Finally, statistically significant enrichment of noisy ultradian rhythms was visible in at least one experiment in liquid media from an independent laboratory (Hurley et al., 2014). This raises the possibility that the ∼7-hr rhythms might be observed only in liquid culture conditions, which are not amenable to many standard assays, such as promoter-based luciferase assays. This might explain why they have not been observed previously.

There were a third as many third harmonic genes as circadian ccgs, which is a much higher fraction than that observed in the mouse liver (63/3,667) (Hughes et al., 2009). Intuitively, we expect to observe successively fewer genes at the higher harmonics if they are derived from the circadian clock, similar to the mouse liver (Hughes et al., 2009). For instance, two appropriately phased circadian TFs can drive second harmonic rhythms (Westermark and Herzel, 2013). However, third harmonic rhythms could be generated by three appropriately phased circadian TFs, which seems harder to achieve. Our discovery of many more third harmonic than second harmonic rhythms is hence even more remarkable.

In addition to being expressed in two anti-phasic clusters (Groups 1 and 2), the third harmonic genes in each cluster also shared highly similar expression profiles. In fact, this discordant co-regulation of Group 1 and Group 2 genes appeared to be the most robust feature across all the genotypes we studied, including in the independent study from Hurley et al. (2014). This suggests that all the third harmonic genes within a cluster might be regulated by a common mechanism. We found significant enrichment of only one TF, called female fertility 7 (FF-7). FF-7, a putative O-acetyl transferase, had a third harmonic transcript, and about a third of the third harmonic genes had a binding site for FF-7. However, FF-7 did not have a preference for either Group 1 or Group 2 genes. This suggests that FF-7 might be a possible candidate common regulator that transmits the harmonic signal to downstream genes. In fact, FF-7 targets itself (Table S1), and this auto-regulatory feedback could be the involved in third harmonic generation.

We hypothesized that the circadian clock might drive harmonics via circadian TFs binding to the promoters of the third harmonic genes. We did not, however, find any (statistically significant) evidence in the available ChIP-seq data for binding of known circadian TFs in the vicinity of harmonic genes. Moreover, the knockout of one circadian repressor, CSP1, did not abolish the third harmonic rhythms, but rather superimposed circadian rhythms on these genes. This strongly suggests that at least a part of the regulation might be post-transcriptional. Post-transcriptional circadian regulation is widespread in *Neurospora* (Hurley et al., 2014). Comparison of promoter-based luciferase assay and qPCR for the genes can reveal the contribution of transcriptional and post-transcriptional regulation of the harmonic genes. To test the broader connection between the circadian clock and harmonics, a study of harmonics in the transcriptome of the clock-less *frq*^0^ strain under the same conditions would be valuable.

Based on the KEGG pathway enrichment, we hypothesized and confirmed that MSN1 is important for 7-hr rhythm generation. In particular, knockout of MSN1 transformed the third harmonic rhythms to second harmonic (11 hr) rhythms. This is consistent with one proposed paradigm for harmonic generation, where we expect that three circadian inputs with appropriate phases are necessary for generating third harmonic rhythms. The ΔMSN1 phenotype is suggestive of the loss of one such factor resulting in second harmonic rhythms. Finally, ΔMSN1 phenotype is indicative of the possible contribution of MAPK or stress response signaling to the harmonics. Similarly, endoplasmic reticulum (ER) stress is shown to initiate 12-hr rhythms in the mouse liver (Zhu et al., 2017).

There is yet the argument that sampling immediately after the light-to-dark transition can generate transients, because many genes in *Neurospora* are known to be light inducible (Chen et al., 2009, Sancar et al., 2015a). However, we found very few light-induced genes (even over a span of 120 min) in the third harmonic gene set, and thus light does not seem to play a role through known mechanisms. We did also find significant dampening of the rhythms in the two genes we studied over the longer 46-hr time course, which might be due to loss of coherence within the population or from loss of single-cell rhythms. Regardless, we cannot exclude the possibility that the rhythms are transients.

Another open question is whether the harmonics are generated by an independent clock or an oscillator. There is a rich history of non-circadian oscillations in *Neurospora*, such as the FRQ-less oscillator (FLO) (Sussman et al., 1964), nitrate reductase oscillator (NRO) (Christensen et al., 2004), and choline deficiency oscillator (CLO) (Lakin-Thomas, 1998), that can show periods between 10 and 100 hr depending on metabolic and temperature conditions. Importantly, these non-circadian rhythms have not been observed simultaneously with an intact circadian oscillator as is the case here. In addition, the non-circadian oscillators do not have the hallmarks of clocks, namely, temperature and metabolic compensation of the period. Our analyses do not preclude a circadian-clock-independent mechanism for harmonic generation including an independent oscillator in resonance with the circadian clock resulting in the harmonic period relationship.

The third harmonic genes are functionally involved in every aspect of the information flow within the cell from transcription, translation, post-translational modification, and transport/chaperones and could thus be vital to the organism. Moreover, tricarboxylic acid cycle (in addition to MAPK and stress response signaling) was implicated in the KEGG pathway, raising the possibility that the harmonics arise from one of these fundamental processes. In addition, it appears that *Neurospora* and mice share four families of harmonic genes that appear to be conserved between these two model organisms. Since the mechanistic basis for harmonics is yet unclear in all model organisms, this conservation might stimulate future studies on the underlying mechanism. Moreover, *Neurospora* provides a simpler circadian model system than mammals for studying mechanisms of harmonic generation.

A functional role for these harmonics is difficult to determine, and, in fact, no clear role for harmonics has yet been identified for any transcriptomic, proteomic, or metabolic ultradian rhythms (Hughes et al., 2009, Krishnaiah et al., 2017, Zhu et al., 2017), with the exception of one 12-hr gene, IRE1α. IRE1α plays a role in lipid metabolism in the endoplasmic reticulum in the mammalian liver, and its second harmonic rhythm is circadian clock dependent (Cretenet et al., 2010). One theory is that these rhythms are ultradian slave oscillators that can resonate to inputs from the circadian clock and hence lock onto harmonic period relationships. Such slave oscillators might be used to temporally tune certain processes in the organism and can act as a “playground” for evolution without disturbing the already fine-tuned circadian system.

Ultradian rhythms appear to be also widespread in the physiology and behavior of more complex eukaryotes, including the dopaminergic oscillator driving arousal in the brain (Blum et al., 2015), gene expression (Zhu et al., 2017, Ono et al., 2015), and in hormone secretion (Walker et al., 2012, Tannenbaum and Martin, 1976), which appear to persist on ablation of the suprachiasmatic nucleus in the brain. There is also evidence of ultradian rhythms being relevant to the ecology of organisms, for example, ultradian feeding rhythm of common voles (Daan and Slopsema, 1978) and rhythms in parental care in birds (Bulla et al., 2016).

The coexistence of ultradian and circadian rhythms across a variety of scales and organisms has important bearing on the way we analyze rhythmic empirical data. Most analysis methods assume rhythms with a single mode (peak) of activity over a 24-hr period and disregard both ultradian rhythms and other “non-sinusoidal” periodic waveforms. The latter might indicate a confluence of multiple period inputs, and even irregular waveforms might have biological significance in their own right, a fact also highlighted by Zhu et al. (2017). Future studies would do well to consider different periods and waveforms to construct a complete picture of rhythms over circadian timescales.

## Limitations of the Study

We would like to discuss a few limitations of the study. First, the transcriptomic data used in this study were derived from liquid cultures similar to other transcriptomic datasets on *N*. *crassa*. Most of the research on *Neurospora* as a model organism has been carried out on solid media cultures. It is unclear what the effects of the culture conditions (temperature, media) on the physiology of this organism are and how these results relate to past studies. Second, as a predominantly computational study, the key results, such as the number of harmonic genes and functional categories, are dependent on the choice of threshold, and the significance measures are highly dependent on the choice of null hypothesis. We have attempted to present the results in a way to minimize the *qualitative* effect of choice of thresholds (e.g., number of genes over a range of FDR thresholds).

## Methods

All methods can be found in the accompanying Transparent Methods supplemental file.

## Supplemental Information

Document S1. Transparent Methods, Figures S1–S5, and Tables S3 and S4

Table S1. Circadian and Harmonic Genes Identified in the WT *N. crassa* Strain, Related to Figures 1 and S1 Lists of the harmonic genes identified in the original and verification datasets along with the amplitude and phase of the rhythms and presence of binding sites of known transcription factors in the vicinity of the gene.

Table S2. Functional and KEGG Pathway Enrichment of the Third Harmonic Genes, Related to Figure 1 Assignment of the third harmonic genes to the significant (*p* < 0.05) functional categories identified by FungiFun2. The results of a similar enrichment analysis of third harmonic genes in the KEGG pathways are also shown.
