# Supplementary material for: Ultradian Rhythms in the Transcriptome of Neurospora crassa
Source: iScience. 2018 Nov 10;9:475–86. doi: 10.1016/j.isci.2018.11.012 (PMC6260400; doi:10.1016/j.isci.2018.11.012)
Supplement: Document S1. Transparent Methods, Figures S1–S5, and Tables S3 and S4 [file mmc1.pdf]

ISCI, Volume *9*

Supplemental Information

Ultradian Rhythms in the Transcriptome of *Neurospora crassa*

Bharath Ananthasubramaniam, Axel Diernfellner, Michael Brunner, and Hanspeter Herzel

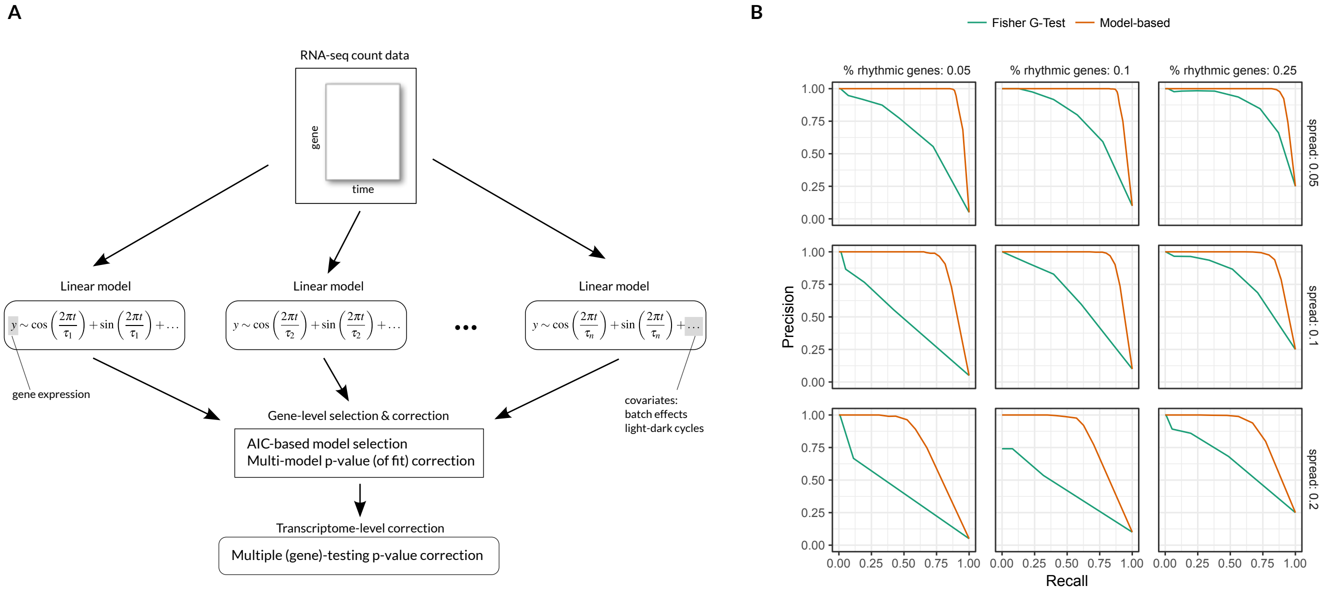

Figure S1: Performance of the model selection-based approach on simulated RNA-seq data, Related to Figure 1 and Transparent Methods. (A) The model selection-based approach for identifying and classifying rhythmic genes into harmonic and circadian genes. The linear model framework with variance modeling at the observational level allows for both modeling the statistical properties of RNA-seq count data and at the same time include other covariates, such as batch effects and light-dark environmental cycles. (B) Comparison of the proposed model selection-based approach and the standard method based on the Fisher G-Test (Wichert et al. 2004) using precision-recall curves. Artificial RNA-seq count data for 5000 genes was simulated under the negative binomial model with different spreads (= 1/size) and different fractions of true positive (rhythmic with periods 22h, 11h or 7.3h) genes to perform this comparison.

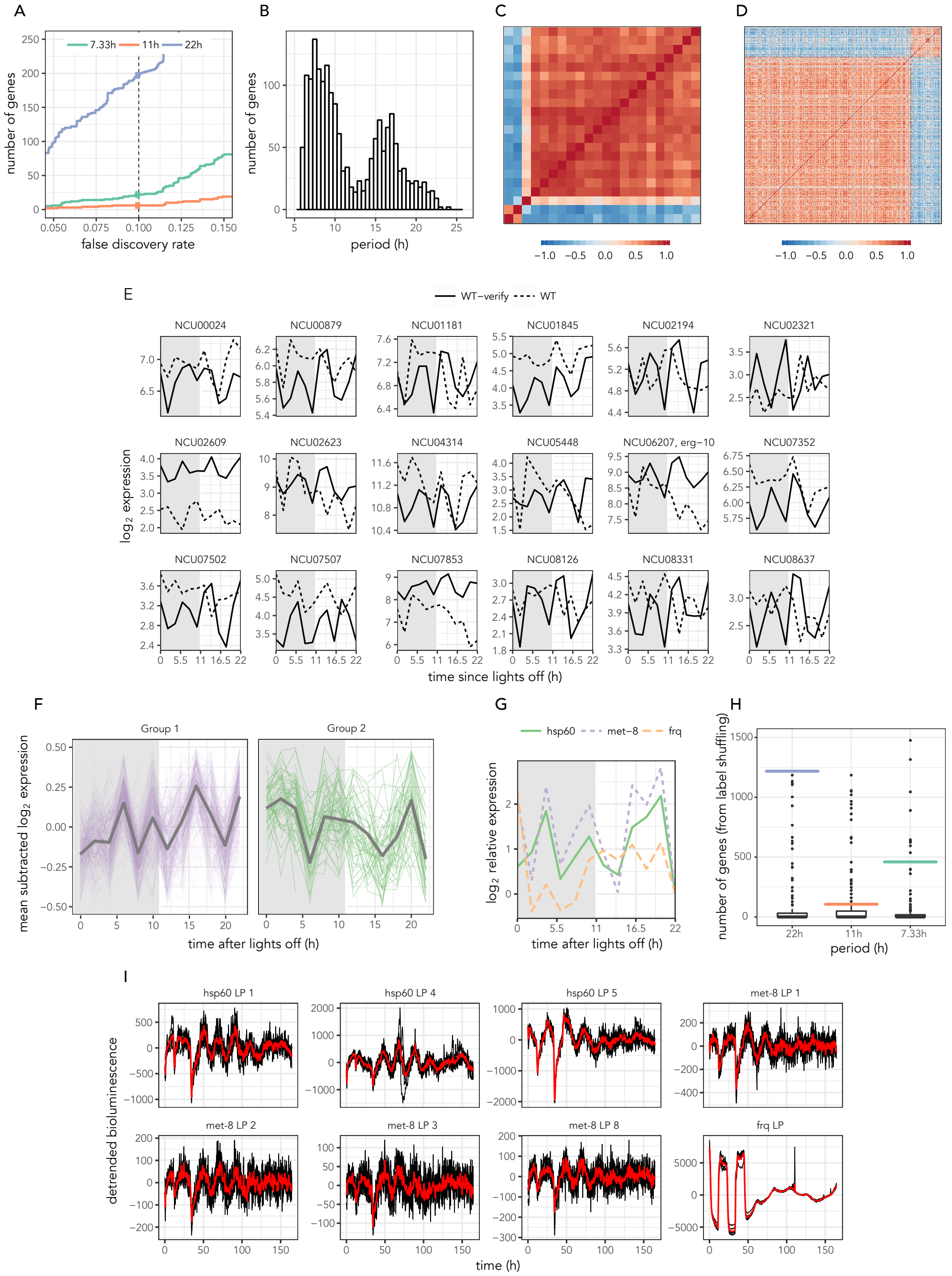

Figure S2: Verification of ultradian rhythms in an independent WT dataset, Related to Figure 1. (A) Model selection-based identification of rhythmic genes in WT *N.crassa* from an independent experiment. Note: lower sequencing depth was available leading to smaller signal-to-noise ratio compared to the original experiment. (B) Confirmation of the result in (A) using an independent ARSER method. (C) Hierarchical clustering of the pairwise expression profiles of the third harmonic genes identified in the independent verification dataset. (D) Pairwise correlation of the expression profiles from the independent experiment clustered according to Group 1 and Group 2 identified in Figure 1. (E,F) Comparison of the expression of selected genes (E) and Group 1 and Group 2 genes (F) in the new WT dataset. The original WT expression profiles are also shown in (E). The mean expression profile of each group is shown in gray in (F). (G) Confirmation of third harmonics in the new WT dataset by qRT-PCR quantification of the selected genes, *hsp60* and *met-8*, with the core clock gene *frq* as the positive circadian control. (H) The number of rhythmic genes at the each harmonic at FDR<0.1 from shuffling the time labels of the WT time course data 10000 times and running our entire pipeline on the shuffled time course. The boxplots represent the distribution of "hits" at each harmonic. The colored lines are the number of genes at each harmonic found in Figure 1 for a FDR<0.1. The estimated empirical false discovery rate from shuffling was 0.07 for all rhythmic genes. (I) Bioluminescence recording of the promoter activity of *hsp60* and *met-8* in selected clones grown in solid media with *frq* promoter as control. Cutures were subjected to two 11h:11h light dark cycles before release into constant darkness of the remainder of the experiment. The black lines are the technical replicates and the red line their average.

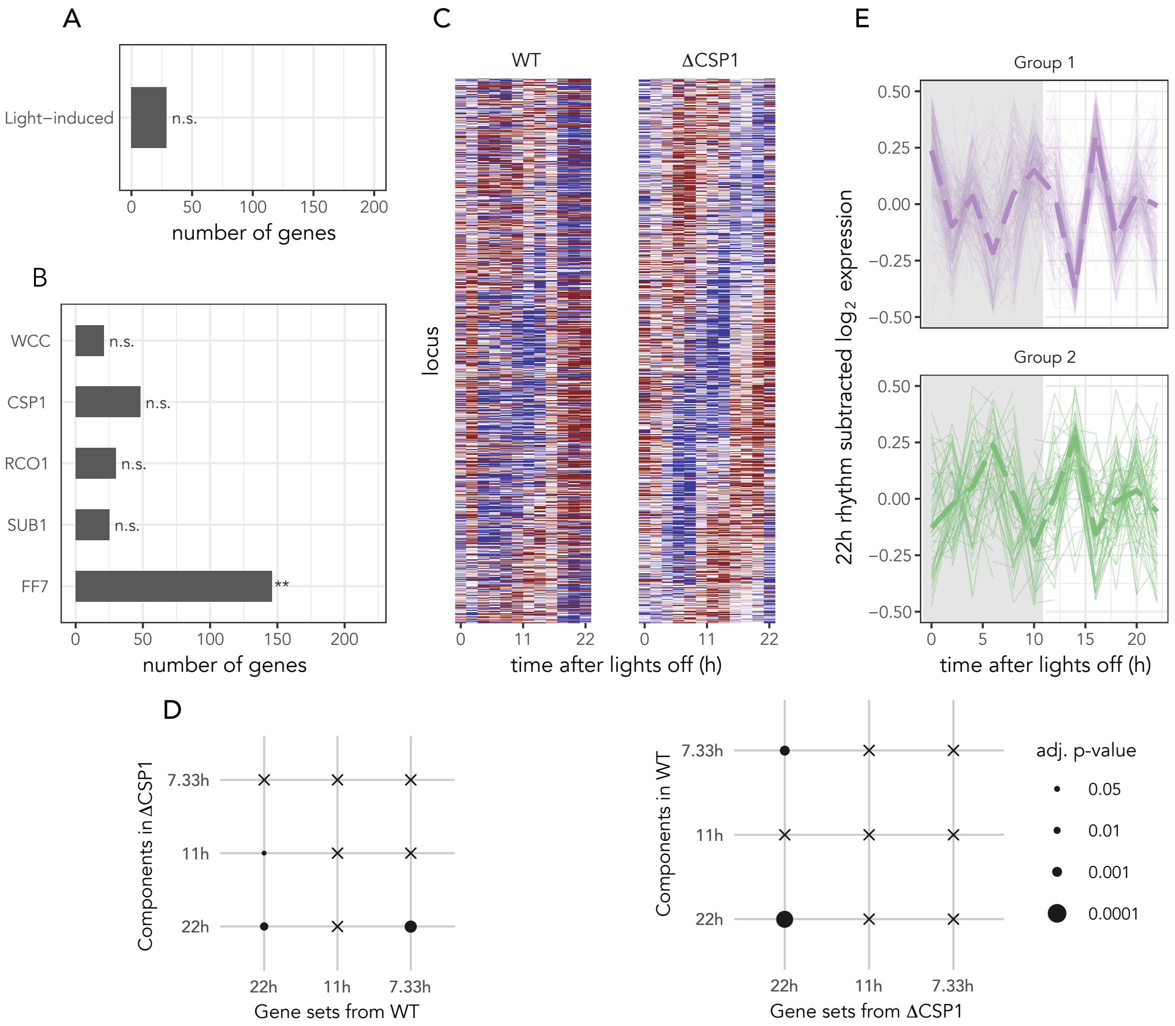

Figure S3: Influence of the circadian clock on third harmonic rhythms, Related to Figures 2 and 3. (A) Functional genome-wide identification of light induced genes in *Neurospora* that also have third harmonic transcripts. (B) Enrichment of the binding of circadian transcription factors in the vicinity of the ∼7h rhythm genes. (C) Comparison of the circadian transcriptome (as defined by 22h period genes in the WT) of the WT and ΔCSP1 strains. (D) Competitive gene set testing (Wu & Smyth 2012) of the gene sets identified as harmonic in the WT for different harmonic components in the ΔCSP1 strain (left) and vice-versa (right). Adjusted p-values are rounded up to the four levels shown and crosses represent no significance (> 0.05). (E) The 22h (circadian) rhythm subtracted expression profiles of the ΔCSP1 strain for the Group 1 and Group 2 genes. The mean expression of each group is displayed in thick dashed lines.

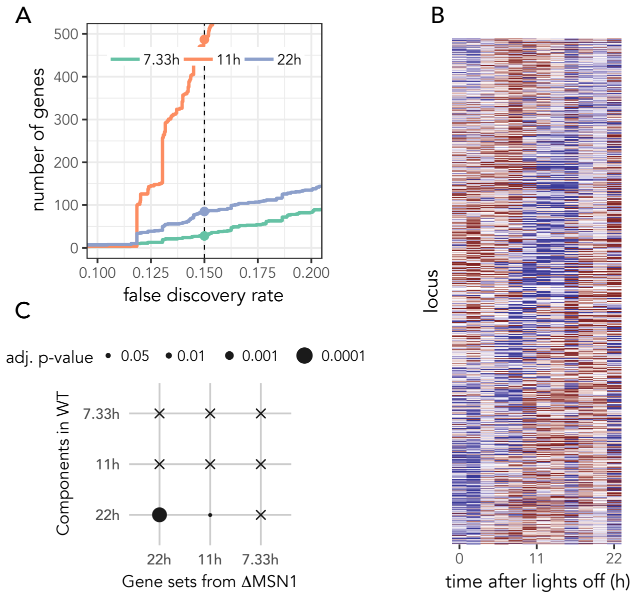

Figure S4: Analysis of harmonics in the ΔMSN1 strain, Related to Figure 4. (A) Model selection-based classification of harmonics in the ΔMSN1 strain. Note the different choice of FDR threshold. (B) The expression of the circadian genes (from the WT) in the ΔMSN1 strain sorted according to phase in the knockout strain. (C) Competitive gene set testing (Wu & Smyth 2012) of the gene sets identified as harmonic in the ΔMSN1 strain for different harmonic components in the WT strain (complement of Figure 4C). Adjusted p-values are rounded up to the four levels shown and crosses represent no significance (> 0.05).

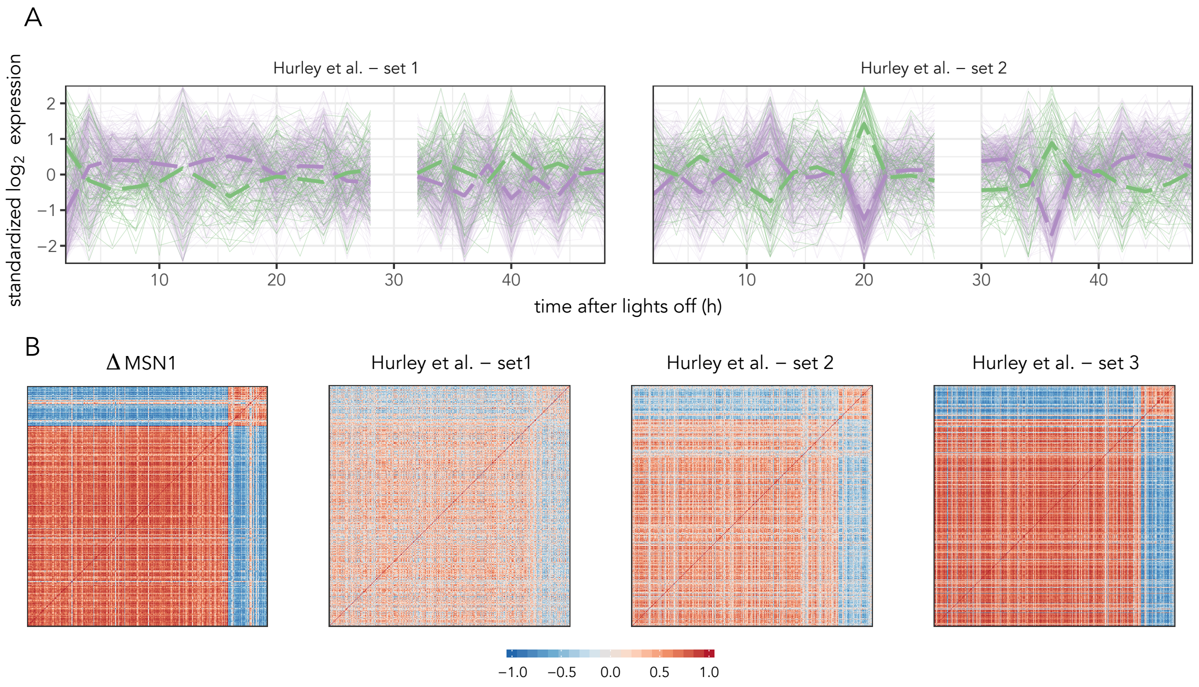

Figure S5: Discordant co-regulation of the two groups of third harmonic genes, Related to Figure 4. (A) The standardized (z-score) expression profiles of the Group 1 (violet) and Group 2 (green) genes in the two remaining time courses in Hurley et al. (2014). The average of the standardized profiles in each group are shown as thick dashed lines. Missing data points were omitted from the plots. (B) Pair-wise correlation of the mean-subtracted gene expression profiles of the third harmonic genes sorted into Group 1 and Group 2 for the different genotypes shown in (A) and Figure 4D.

Table S3: The high throughput RNA-seq and ChIP-seq data used in this study and their essential details, Related to Figures 1, 3 and 4 and Transparent Methods.

**Accession number**	**sequencing type**	**experiment**	**light conditions**	**reference**
SRA: SRX547956	single-end, unstranded	wild-type, time course over 22h every 2h	growth in 11h L: 11h D, collection in DD from L to D transition	Sancar, Sancar, Ha, Cesbron & Brunner (2015)
SRA: SRX547959	single-end, unstranded	CSP1 KO, time course over 22h every 2h	growth in 11h L: 11h D, collection in DD from L to D transition	Sancar, Sancar, Ha, Cesbron & Brunner (2015)
SRA: SRP046458	single-end, stranded	wild-type, time course over 48h every 2h	growth in constant light, collection in DD from L to D transition	Hurley et al. (2014)
GEO: GSE113845	single-end, stranded	wild-type, time course over 48h every 2h	growth in 11h L: 11h D, collection in DD from L to D transition	this work
GEO: GSE113845	single-end, stranded	MSN1 (NCU02671) KO, time course over 22h every 2h	growth in 11h L: 11h D, collection in DD from L to D transition	this work

Table S4: Chromatin-immunoprecipitation (ChIP)-sequencing data used in this study, Related to Figure S3 and Transparent Methods.

**transcription factor**	**condition**	**source**	**reference**
WCC	growth in LL, after 12h dark and 8 min light	Table S1	Smith et al. (2010)
CSP1	growth in DD, after 30 min light exposure	Table S1	Sancar et al. (2011)
RCO-1	growth in DD, after 30 min light exposure	Table S1	Sancar et al. (2011)
FF-7	growth in LL	Table S4	Sancar, Ha, Yilmaz, Tesorero, Fisher, Brunner & Sancar (2015)
SUB-1	growth in dark or light	Table S1	Sancar, Ha, Yilmaz, Tesorero, Fisher, Brunner & Sancar (2015)

Transparent Methods

Data and Software Availability

Most datasets used in this study were previously published with data submitted to short read archive (SRA) and the protocols for preparation of the library can be found in the respective publication. The accession number for the sequencing data reported in this paper is GEO: GSE113845. These two novel datasets were collected following the exact protocol in (Sancar, Sancar, Ha, Cesbron & Brunner 2015) (SRA: SRX547956) under wild-type and ΔMSN1 genetic backgrounds, respectively. Table S3 summarizes the essential parameters for these datasets. The publicly-available ChIP-seq data used in this study are listed in Table S4.

RNA sequencing analysis

The sequencing data was summarized at the transcript-level using the pseudoalignment approach of *kallisto* (v 0.43.1) (Bray et al. 2016) based on the *Neurospora* NC12 genome assembly. Gene level counts from scaled TPM values and the effective transcript lengths were computed from transcript-level estimates using the *tximport* R package (Soneson et al. 2015). The count data were then subsequently analyzed using the linear modeling approach with mean-variance relationship estimation of the *limma-voom* R package (Law et al. 2014). We retained only the 'expressed' genes for analysis that we define as any gene that had at least 10-15 raw counts in at least 80% of each time series. All analyses were performed using R (version 3.4.1).

Identification of harmonics

We used a model selection approach to classify each gene expression time series as having either a 22h, 11h or 7.33h rhythm – since *N. crassa* has an intrinsic period of $\sim$ 22.5h, we set 22h as the circadian period length. We used the linear model formulation with variance modeling at the observational level available under *limma-voom* (Law et al. 2014). We first fit sample specific weights (Liu et al. 2015) using a model with all three harmonics and then used these array weights to fit rhythms of each period separately. Only $a\cos(2\pi t/T) + b\sin(2\pi t/T)$ needs to be fit to each gene for each period T, which is linear regression problem. Although our approach allowed it, we did not include any batch-effects in our models. The best fitting model of the three is selected using the Akaike Information Criterion (using `selectModel` function in *limma*) and the p-value for the best fitting model is obtained from the F-test (from `lmFit`, `eBayes` and `topTable`) after correcting for multiple testing using Benjamini-Hochberg (BH) (since we compare three different models). Once the best-fitting model for each gene is available, the p-values are again corrected using BH for the multi-gene comparison (overview in Figure S1A). Finally, the hits are identified as genes with corrected p-value (false discovery rate) < 0.1 and amplitude of oscillation under the best-model of 1.5-fold peak-to-trough amplitude (effect size threshold). The advantage of this approach is that we can account for the underlying statistical properties of the RNA-sequencing data and simultaneously also include other covariates of gene expression, such as batch effects. Our proposed approach had significantly better precision-recall performance in comparison to the standard method used to identify harmonics based on the Fisher-G Test (Wichert et al. 2004) (Figure S1B).

ChIP-seq data and analysis

We used the significant bindings peaks identified in the respective studies that were provided in the supplementary tables (see Table S4 for details). We considered proximal genes both upstream and downstream of the identified TF binding sites as valid targets.

Gene set enrichment analysis

In order to find overlaps in the genes identified in two different experiments, we resorted to correlation-corrected mean rank set (*camera*) test (Wu & Smyth 2012). Gene set tests are statistically superior to simple overlaps of 'hits' from both experiments. In particular, we looked for enrichment of the specific harmonic component in the combined fit of all three periods (22h, 11h and 7.33h) in one dataset (test set) to the gene sets identified in the other (reference set). The bidirectional overlap can be obtained by repeating the procedure by exchanging the reference and test sets. The p-values from these multiple tests were then multiple testing corrected using the Benjamini-Hochberg procedure.

Plasmid construction and *Neurospora* transformation

The *met-8* promoter (NCU06512, ca. 1600 bp) and the *hsp60* promoter (NCU01589, ca. 1500 bp) were amplified by PCR and inserted into via EcoRI/NotI, into pFH62 lucPEST (Cesbron et al. 2013). Primer sequences were: pNCU01589 F: `5'-cccccgaattcAAAAGTCGAGTCTTTGAGGCG-3'`, pNCU01589 R: `5'-aaaaagcggccgcAACTGGGGAAGAAAAGTGCG-3'`, pNCU06512 F: `5'-cccccgaattcAGAGGAAGTTTCCTTCGTAGG-3'` and pNCU06512 R: `5'-aaaaagcggccgcGTGACCTAGTCTGATTTTCGG-3'`. Neurospora conidia were transformed as described (Schafmeier et al. 2006). The Neurospora strains carried the *ras-1*^{bd} mutation (Belden et al. 2007). For transformations, *ras-1*^{bd}; *his-3* (Aronson et al. 1994) was used. Construction of the strain expressing lucPEST under the control of the frq promoter is described elsewhere (Cesbron et al. 2013).

In vivo Luciferase Measurements

Solid sorbose medium containing 1× FGS (0.05% fructose, 0.05% glucose, 2% sorbose), 1× Vogels, 1% agarose, 10 ng/ml biotin and 75 μM firefly luciferin was used for the assessment of the luciferase rhythms. 96-well plates were inoculated with 3×10^4 conidia per well and incubated in light at 25 °C for 2 days. Bioluminescence was then recorded with an EnSpire Multilabel Reader (Perkin Elmer) every 30 min for 44 h at 25 °C under LD 11h:11h before release into constant darkness for the remainder of the measurement. Light intensity used in LD cycles was 40 μE.

Neurospora circadian time course

Liquid cultures were inoculated on two sets of sealed petri dishes supplied with standard growth medium (2% glucose, 0.5% L-arginine, 1× Vogel's) and incubated without shaking for 3 days at 25 °C in LD 11h:11h to grow mycelial mats; one set of petri dishes phase shifted 11h to the other. Mycelial discs were then punched out at the transition from L to D and D to L, respectively, and transferred to shaking 500 ml flasks containing 150 ml of standard growth medium under the continuing LD regime. At the next respective LtoD transition the cultures were released into constant darkness and subsequently timepoints DD 0-10 and DD 12-22 were harvested in 2 h intervals the following day. 24 hours later timepoints DD

24-34 and DD 36-46 were harvested. RNA extraction, cDNA preparation and qRT-PCR were performed as described below.

qRT-PCR

RNA was extracted from powdered mycelia using peqGOLD TriFAST (peqLab, Erlangen, Germany) according to the manufacturer's protocol. Precipitated RNA was dissolved in 100 μl nuclease free water supplied with 80u Ribolock RNAse inhibitor (ThermoScientific, Waltham, MA US). Maxima First Strand cDNA Synthesis Kit (ThermoScientific, Waltham, MA US) was used for cDNA synthesis. Transcript levels were analysed by qRT-PCR in 96-well plates with the StepOnePlus Real-Time PCR System (Life Technologies, NY, USA) using qPCRBIO Probe Mix Hi-Rox (PCR Biosystems Ltd, London, UK). Samples were measured in triplicates and evaluated using the $\Delta\Delta$CT method normalising to 28S rRNA with the timepoint with minimum value set to 1. The following primers and probes were used: hsp-60 (NCU06512): F: `ccgtcctcgtcttcgatct`, R: `aagtggcaacgacgacct`, probe: UPL #9 (Universal Probe Library, Roche); met-8 (NCU01589): F: `cgacgtcaagctcgagaag`, R: `atgatggtgtcgtccttggt`, probe UPL #9; frq (NCU02265): F: `ttgtaatgaaaggtgtccgaaggt`, R: `ggaggaagaagcggaaaacg`, probe: 6 FAM `acctcccaatctccgaactcgcctg` TAMRA; 28S rRNA (used for normalization): F: `gaacaacagggattgcccta`, R: `ggactcagaaggtgcctcac`. probe: 6 FAM `tgaaatctggcttcggcccg` TAMRA.

Supplemental References

Aronson, B. D., Johnson, K. A. & Dunlap, J. C. (1994), 'Circadian clock locus frequency: protein encoded by a single open reading frame defines period length and temperature compensation.', *Proceedings of the National Academy of Sciences* **91**(16), 7683–7687.

Belden, W. J., Larrondo, L. F., Froehlich, A. C., Shi, M., Chen, C.-H., Loros, J. J. & Dunlap, J. C. (2007), 'The band mutation in Neurospora crassa is a dominant allele of ras-1 implicating RAS signaling in circadian output', *Genes & Development* **21**(12), 1494–1505.

Bray, N. L., Pimentel, H., Melsted, P. & Pachter, L. (2016), 'Near-optimal probabilistic RNA-seq quantification', *Nat Biotech* **34**(5), 525–527.

Cesbron, F., Brunner, M. & Diernfellner, A. C. R. (2013), 'Light-Dependent and Circadian Transcription Dynamics In Vivo Recorded with a Destabilized Luciferase Reporter in Neurospora', *PLoS ONE* **8**(12), e83660.

Hurley, J. M., Dasgupta, A., Emerson, J. M., Zhou, X., Ringelberg, C. S., Knabe, N., Lipzen, A. M., Lindquist, E. A., Daum, C. G., Barry, K. W., Grigoriev, I. V., Smith, K. M., Galagan, J. E., Bell-Pedersen, D., Freitag, M., Cheng, C., Loros, J. J. & Dunlap, J. C. (2014), 'Analysis of clock-regulated genes in Neurospora reveals widespread posttranscriptional control of metabolic potential', *Proceedings of the National Academy of Sciences* **111**(48), 16995–17002.

Law, C. W., Chen, Y., Shi, W. & Smyth, G. K. (2014), 'voom: precision weights unlock linear model analysis tools for RNA-seq read counts', *Genome Biology* **15**(2), R29.

Liu, R., Holik, A. Z., Su, S., Jansz, N., Chen, K., Leong, H. S., Blewitt, M. E., Asselin-Labat, M.-L., Smyth, G. K. & Ritchie, M. E. (2015), 'Why weight? Modelling sample and observational level variability improves power in RNA-seq analyses', *Nucleic Acids Research* **43**(15), e97–e97.

Sancar, C., Ha, N., Yilmaz, R., Tesorero, R., Fisher, T., Brunner, M. & Sancar, G. (2015), 'Combinatorial Control of Light Induced Chromatin Remodeling and Gene Activation in Neurospora', *PLOS Genetics* **11**(3), e1005105.

Sancar, C., Sancar, G., Ha, N., Cesbron, F. & Brunner, M. (2015), 'Dawn- and dusk-phased circadian transcription rhythms coordinate anabolic and catabolic functions in Neurospora', *BMC Biology* **13**, 17.

Sancar, G., Sancar, C., Brügger, B., Ha, N., Sachsenheimer, T., Gin, E., Wdowik, S., Lohmann, I., Wieland, F., Höfer, T., Diernfellner, A. & Brunner, M. (2011), 'A Global Circadian Repressor Controls Antiphasic Expression of Metabolic Genes in Neurospora', *Molecular Cell* **44**(5), 687–697.

Schafmeier, T., Kaldi, K., Diernfellner, A., Mohr, C. & Brunner, M. (2006), 'Phosphorylation-dependent maturation of Neurospora circadian clock protein from a nuclear repressor toward a cytoplasmic activator', *Genes & Development* **20**(3), 297–306.

Smith, K. M., Sancar, G., Dekhang, R., Sullivan, C. M., Li, S., Tag, A. G., Sancar, C., Bredeweg, E. L., Priest, H. D., McCormick, R. F., Thomas, T. L., Carrington, J. C., Stajich, J. E., Bell-Pedersen, D., Brunner, M. & Freitag, M. (2010), 'Transcription Factors in Light and Circadian Clock Signaling Networks Revealed by Genomewide Mapping of Direct Targets for Neurospora White Collar Complex', *Eukaryotic Cell* **9**(10), 1549–1556.

Soneson, C., Love, M. I. & Robinson, M. D. (2015), 'Differential analyses for RNA-seq: transcript-level estimates improve gene-level inferences', *F1000Research* **4**, 1521.

Wichert, S., Fokianos, K. & Strimmer, K. (2004), 'Identifying periodically expressed transcripts in microarray time series data', *Bioinformatics* **20**(1), 5–20.

Wu, D. & Smyth, G. K. (2012), 'Camera: a competitive gene set test accounting for inter-gene correlation', *Nucleic Acids Research* **40**(17), e133–e133.
